# Flavor, Lipid, and Transcriptomic Profiles of Chinese Wagyu Beef Cuts: Insights into Meat Quality Differences

**DOI:** 10.3390/foods14050716

**Published:** 2025-02-20

**Authors:** Tianliu Zhang, Tingting Wang, Yanhao Gao, Jiashun Sheng, Hossam E. Rushdi, Wentao Li, Yu Sun, Tong Fu, Feng Lin, Tengyun Gao, Shenhe Liu

**Affiliations:** 1 College of Animal Science and Technology, Henan Agricultural University, Zhengzhou 450046, China; zhangtianliu@henau.edu.cn (T.Z.); 16696736731@163.com (T.W.); gaoyanhao_eureka@163.com (Y.G.); 13419966839@163.com (J.S.); nongdaliwentao@163.com (W.L.); sunyu95@163.com (Y.S.); futong2004@126.com (T.F.); linfeng7207@163.com (F.L.); 2 Department of Animal Production, Faculty of Agriculture, Cairo University, Giza 12613, Egypt; hosamrushdi@agr.cu.edu.eg

**Keywords:** Chinese Wagyu cattle, beef cuts, metabolites, gene expression, lipid

## Abstract

This study aimed to investigate the flavor formation and meat quality differences among different beef cuts in Chinese Wagyu cattle. The metabolites and gene expression profiles of chuck, neck, rump, tenderloin, and longissimus lumborum cuts were analyzed. The results revealed that a total of 240 volatile organic compounds and 779 lipid molecules were detected among the beef cuts, with hydrocarbons (accounting for 29.71%) and triglycerides (representing 41.21%) emerging as the most prominent compounds, respectively. The sensory-directed analysis highlighted the significance of sweet and fruity aroma compounds, which contributed to the distinct aroma profiles among different beef cuts. Additionally, a total of 60 key lipid molecular markers, including FA(18:1), PC(40:5), TG(18:0_16:1_18:1), and TG(36:0_18:1), etc., were identified as playing crucial roles in the generation of essential lipid compounds across five different beef cuts. Integrative analysis of multi-omics data pinpointed a cluster of differentially expressed genes (e.g., *DLD*, *ACADM*, *PCCA*, *SCD*), which were involved in the regulation of valine, leucine, and isoleucine degradation pathways and lipid metabolism. Taken together, this study has identified key metabolites and candidate genes influencing meat quality across different beef cuts, providing a valuable resource for the molecular breeding of high-quality traits in beef cattle.

## 1. Introduction

Beef is the third most consumed meat worldwide and can provide essential nutrients for human health. With the improvement in people’s consumption levels, the nutritional quality of meat has received remarkable attention, since the meat flavor affects consumer preferences in recent years [1]. Beef quality is influenced by various factors, such as age, breed, marbling, flesh color, tenderness, pH, intramuscular fat content (IMF), and water-binding capacity [2]. Previous studies have demonstrated that a higher IMF content enhances the flavor of meat, whereas a lower IMF content weakens the flavor precursors in the meat [3,4,5]. The lipid profile in IMF plays a crucial regulatory role as a flavor precursor of meat [6]. During meat processing, these flavor precursors produce volatile compounds with different abundances and compositions through lipid oxidation and the Maillard reaction, which determines the final flavor of the meat [7]. Basically, beef carcasses are classified into various cuts, which include longissimus lumborum, chuck, tenderloin, etc. These beef cuts have natural physiological functions and nutritional components that affect meat quality [8].

Beef cuts from different carcass regions may contain different chemicals, lipids, metabolites, and types of muscle fiber, resulting in distinct flavor characteristics. The proportion of type I fibers in tenderloin is higher compared to that in longissimus lumborum, resulting in the meat having a lower fat content and reduced tenderness [9]. Meanwhile, with increasing acid discharge duration, the mitochondrial concentration and mitochondrial oxygen consumption for the tenderloin muscle showed a trend of change from high to low [10]. Advanced high-throughput sequencing techniques have facilitated a comprehensive understanding of large-scale profiles of small molecule metabolites. Recently, several studies on beef meat quality characteristics have been performed using multi-omics approaches. Yu et al. compared the muscle-specific beef quality between the tenderloin and longissimus lumborum of the Chinese Jinjiang yellow cattle, and identified important oxidative phosphorylation signaling pathways as well as detected several candidate genes (*SLC16A7*, *HOXC6*, and *HOXC8*) [11]. Using pasture-finished Nellore bulls, Giovanini et al. assessed the dynamics of the lipidomic and metabolomic profiles for longissimus lumborum at different ultimate pH degrees and postmortem aging times. The authors found that C12:0 and C14:0 acylcarnitines were significantly associated with lipid catabolism, suggesting their potential use as biomarkers for meat quality [12]. Interestingly, our previous studies revealed the expression changes and region-specific expression patterns in various beef cuts, and identified 38 and 91 potential candidate genes related to fatty acid composition and meat quality, respectively [13,14]. However, elucidating the potential meat quality differences among different beef cuts using multi-omics data has not yet been well explored.

The objective is to investigate the key metabolites and genes associated with flavor formation and meat quality differences among different beef cuts in cattle. The chuck, neck, rump, tenderloin, and longissimus lumborum cuts of Chinese Wagyu cattle were collected in this study. The flavor compounds, lipid molecules, and gene expression profiles of different beef cuts were analyzed by GC-MS, LC-HRMS, and RNA-seq, and key metabolites and candidate genes were screened. This study provided valuable information for the molecular breeding of meat quality in cattle.

## 2. Materials and Methods

### 2.1. Animal Preparation and Sample Collection

A total of 24 female Chinese Wagyu cattle were obtained from Zhonghe Muxing Food (Langfang) Co., Ltd., Langfang, China. All animals were moved to Pingdingshan Ruibao Red Beef Industry Co., Ltd. (Pingdingshan City, China) for slaughter. During the slaughtering process, six female Chinese Wagyu cattle of similar genetic background, feeding regime, and weight (589 ± 50 kg) were selected for sample collection. Chuck (chuck roll), neck, rump (top round), tenderloin, and longissimus lumborum cuts were frozen in liquid nitrogen. Moreover, meat samples from five different beef cuts were collected and stored for 48 h, after which the samples were vacuum-packed and chilled at −80 °C. All animal procedures were conducted according to the guidelines specified for animal protection and welfare by the Ministry of Agriculture of China. Beef cut samples were collected with the approval of the Ethics Committee of the Science Research Department of the Institute of Animal Science, Henan Agricultural University, under HMUD2024031224.

### 2.2. Analyses of the Volatile Organic Compounds

The chuck, neck, rump, tenderloin, and longissimus lumborum samples were ground into powder in liquid nitrogen, respectively. Around 0.2 g of powder was immediately transferred to a 20 mL headspace vial (Agilent Technologies, Palo Alto, CA, USA), containing 0.2 g of NaCl powder to inhibit enzymatic reactions. For solid-phase microextraction (SPME) analysis, each vial was heated at 60 °C for 5 min, after which a 120 µm PDMS/CWR fiber (Agilent Technologies, Palo Alto, CA, USA) was placed over the sample’s headspace for 15 min at 60 °C. The quality control samples for flavor analysis were prepared by mixing equal numbers of each individual sample. After sampling, the volatile organic compounds (VOCs) were desorbed from the fiber coating in the injection port of the gas chromatography (GC) instrument (Model 8890; Agilent Technologies, Palo Alto, CA, USA). The VOCs were identified and quantified using an Agilent Model 8890 GC instrument and a 7000D mass spectrometer (Agilent) equipped with a 30 m × 0.25 mm × 0.25 μm DB-5MS capillary column. Helium was used as the carrier gas at a linear velocity of 1.2 mL/min, with the injector temperature maintained at 250 °C and the detector temperature set at 280 °C. The oven temperature program was as follows: an initial temperature of 40 °C held for 3.5 min, then increased to 100 °C at a rate of 10 °C/min, followed by an increase to 180 °C at a rate of 7 °C/min, and finally increased to 280 °C at a rate of 25 °C/min, where it was held for 5 min. Mass spectrometry was performed using an electron ionization (EI) source with an ionization energy of 70 eV. The ion source, quadrupole, and transfer line temperatures were set at 230 °C, 150 °C, and 280 °C, respectively. The mass spectrometer operated in selected ion monitoring (SIM) mode for the identification and quantification of analytes. VOCs were identified by comparing their retention indices and drift times with those of standard compounds in the NIST library and the GC-IMS database from GAS. Meanwhile, the relative quantitative analysis was performed based on the peak intensities of the individual volatile flavor compounds. Using the prcomp function in R language, unsupervised principal component analysis (PCA) was conducted to visualize the beef cut samples. The Pheatmap package was utilized to perform the hierarchical clustering of these samples. Variable importance in projection (VIP) values were identified from orthogonal partial least squares-discriminant analysis (OPLS-DA) results [15]. Differential volatile organic compounds (DVOCs) between groups were determined by VIP ≥ 1, *p* < 0.05, and fold change (FC) ≥ 2 or FC ≤ 0.5. Radar and network maps of the sensory flavor characteristics of the DVOCs were plotted using the fmsb and igraph R packages (v.4.3.3), respectively.

### 2.3. Untargeted Lipidomic Analysis

Lipidomic profiles of beef cut samples were performed using untargeted lipidomic with an ultraperformance liquid chromatography (UPLC; Vanquish)–Orbitrap mass spectrometry (MS) system based on the previously reported method [16]. Briefly, 0.5 mg of meat sample was added to a hydrolysis tube with 200 μL of H_2_O. Then, 400 μL of methyl tertbutyl ether (MTBE) and 80 μL of methanol (MeOH) were added, and the mixture was vortexed for 30 s and sonicated for 10 min. The mixture was then centrifuged for 15 min at 3000 rpm to separate the phases, and a white precipitate appeared at the interface. The dried extract was redissolved in 100 μL of DCM:MeOH (1:1, *v*/*v*), and 5 μL of each sample was collected to generate a pooled quality control sample. The UPLC analysis of the lipid extracts was conducted with the following specifications: a Waters HSS T3 column measuring (100 × 2.1 mm, 1.8 μm); a column temperature maintained at 40 °C, a flow rate of 0.3 mL/min, an injection volume of 2 μL, and a solvent system comprising acetonitrile:water (6:4, with 10 mM HCOONH4) and acetonitrile:isopropanol (1:9, with 10 mM HCOONH4). Gradient elution was performed as follows: 70:30 *v*/*v* of the two mobile phases from 0–4 min, 0:100 *v*/*v* from 4.0–22.0 min, and 70:30 *v*/*v* from 22.1–26.0 min.

Liquid chromatography-high-resolution mass spectrometry (LC-HRMS) was conducted on a Q Exactive hybrid quadrupole Orbitrap mass spectrometer (Thermo Fisher Scientific, Waltham, MA, USA) using full-scan MS2 acquisition methods. The parameters for the electrospray ionization (ESI) source were configured as follows: a spray voltage of 3.0 kV/–2.8 kV, a sheath gas pressure of 60 arb, an aux gas pressure of 10 arb, a sweep gas pressure of 0 arb, a capillary temperature of 320 °C, and an aux gas heater temperature of 350 °C. The data were acquired on the Q-Exactive platform using Xcalibur 4.1 (Thermo Fisher Scientific, Waltham, MA, USA) and processed using LipidSearch (Thermo Fisher Scientific, Waltham, MA, USA). Moreover, the precursor ion tolerance was 5 ppm, the product ion tolerance was 5 ppm, and the product ion threshold was 5%. The relative quantities of lipid molecules were calculated by comparing their relative peak areas.

A supervised OPLS-DA of lipidomic data was conducted using the ropls package in R language [17]. Differential lipid molecules (DLMs) were identified by calculating VIP, combined with the T-test and fold change, with the screening threshold set at VIP values ≥ 1, *p*-values < 0.05, and FC ≥ 2 or FC ≤ 0.5. A receiver operating characteristic curve (ROC) was used to select potential lipid markers among the five beef cuts in MetaboAnalyst. Functional enrichment analysis of the DLMs was conducted using the Kyoto Encyclopedia of Genes and Genomes (KEGG) database.

### 2.4. RNA Library Preparation and Sequencing Analysis

Total RNA extraction and mRNA library construction were described according to our previous study [18]. The raw reads were generated per sample using the Illumina Nova Seq 6000 system by paired-end strategy. RNA library construction and sequencing were conducted by Jiangsu Sanzhu Biotechnology Co., Ltd., Nanjing China. To obtain high-quality clean reads and ensure the accuracy of subsequent bioinformatic analysis, FASTP software (v.0.24.0 [19] was used with default parameters to filter and discard reads containing more than 10% ploy-N, trim adaptors, and low-quality reads with a mass value of less than 20. Next, HISAT2-build software (v.2.1.0) [20] was used to construct the index of the ARS-UCD2.0 reference genome. The clean reads were mapped using HISAT2 [20]. SAMtools (v.1.9) was used to sort and convert the SAM files into BAM format [21]. StringTie software (v.1.5.2) was used to estimate the expression levels as fragments per kilobase of transcript per million mapped reads (FPKM) for the transcripts and genes [22]. Considering the effects of biological and technological confounding factors, we retained genes that presented an FPKM value greater than 1 across the six biological replicate samples. To assess the inherent repeatability and determine outliers among the muscle samples, PCA was employed for clustering and visualization of the five types of beef cuts. Hierarchical clustering of these samples was then conducted using the Pheatmap package (v.1.0.12).

Differential expression analysis was performed using the DESeq2 package [23]. Differentially expressed genes (DEGs) were identified with the threshold values of adjusted *p*-value (*p* adj) < 0.05 and |log_2_FC| ≥ 0.58. The UpSetR package was used to visualize the intersections of DEGs among the five types of beef cuts [24]. The DEGs were displayed in a volcano plot using the ggpubr package. We finally performed KEGG pathway enrichment analysis of the DEGs using the Database for Annotation, Visualization, and Integrated Discovery (DAVID), with a significant enrichment threshold of FDR < 0.05 [25].

### 2.5. Statistical Analysis

Statistical analyses were performed using the cited packages in R language. *p* values were determined by one-way ANOVA, Dunnett’s multiple comparison test for five different beef cuts, and Student’s *t*-test for groups. Asterisks signify different significance levels (* *p* ≤ 0.05, ** *p* ≤ 0.01, and *** *p* ≤ 0.001).

## 3. Results

### 3.1. Summary Statistics of Multiple-Omics Dataset

To explore the flavor formation and meat quality changes among diverse beef cuts, we collected five typical beef sections, including chuck, neck, rump, tenderloin, and longissimus lumborum, which were obtained from Chinese Wagyu cattle. A total of 240 VOCs were identified by performing GC-MS analyses. These VOCs were divided into 14 categories according to chemical class, including amines, alcohols, aromatics, nitrogen compounds, sulfur compounds, halogenated hydrocarbons, aldehydes, acids, terpenoids, hydrocarbons, ketones, heterocyclic compounds, esters, and others (Supplementary Dataset S1). We also identified 779 lipid molecules, including 731 in positive ion mode and 48 in negative ion mode (Supplementary Dataset S2). Meanwhile, RNA-seq analysis was performed to ascertain the genes potentially implicated in the regulation of meat quality. After performing rigorous quality control, a total of 1483 million clean paired-end reads (~212 GB) were generated, averaging 49 million reads per sample (Supplementary Table S1). The average read mapping rate was ~94.03%, with a range of 89.88 to 95.96%. The gene expression levels were quantified using the FPKM values, resulting in a total of 9133 genes with reliable expression levels (Supplementary Dataset S3).

### 3.2. Comparative Flavoromic Analysis Among Different Beef Cuts

**Figure 1:**
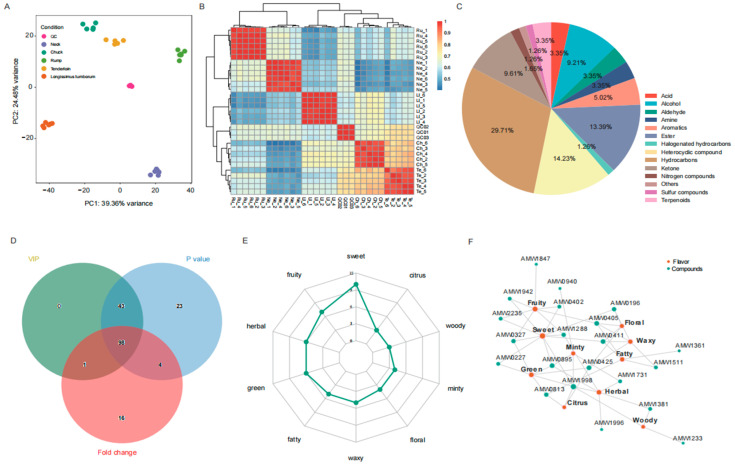
The profile of flavor compounds in the five beef cuts. (**A**) PCA of flavor mass spectrometry data for quality control samples and all beef cuts. (**B**) Unbiased hierarchical clustering heat map for quality control samples and all beef cuts. Both the ordinate and coordinates are labeled with names, where Ll, Ch, Ru, Te, and Ne represent longissimus lumborum, chuck, rump, tenderloin, and neck beef cuts, respectively. (**C**) Percentages of volatile organic compounds based on chemical taxonomy: class. (**D**) Differential volatile organic compounds (DVOCs) were detected by the Venn diagram based on VIP value, *p*-value, and fold change. (**E**) Radar map of DVOCs sensory flavor characteristics. The outermost name represents the sensory flavor feature, and the number corresponding to the green dot represents the number of the corresponding sensory flavor feature. (**F**) Network diagram of sensory flavor characteristics and DVOCs between chuck vs. neck. Orange circles represent sensory flavor characteristics, and turquoise circles represent differential metabolites.

To initially assess the dynamic change in flavorsome profiles, we investigated the VOCs among different beef cuts. PCA of the flavoromic data revealed that the first two principal components were able to explain 63.84% of the total variance, which accounted for 39.36% and 24.48% of the variance, respectively (Figure 1A). The hierarchical clustering analysis showed that the rump and neck samples were located apart from other samples, indicating different flavor characteristics among the five different beef cuts (Figure 1B). To analyze the VOCs responsible for the differences in odor, the quantitation and proportions of 240 VOCs were determined, as shown in Figure 1C. Among the five different beef cut samples, hydrocarbons were the most abundant compounds, accounting for 29.71% of the total volatiles. Heterocyclic compounds and esters followed, accounting for 14.23% and 13.39% of the total, respectively.

To further explore potential VOC changes among beef cuts, we constructed the OPLS-DA model and estimated the VIP between groups. The average values of R2X, R2Y, and Q2 predicted by the OPLS-DA model were 0.718, 0.986, and 0.853, respectively (Supplementary Table S2), confirming that the model was not overfitted and the results were reliable. Under the conditions of VIP ≥ 1, *p* < 0.05, and fold change (FC) ≥ 2 or FC ≤ 0.5, we obtained a total of 139 differential volatile organic compounds (DVOCs) among ten groups, ranging from ten DVOCs in the chuck and tenderloin group to 102 in the longissimus lumborum and neck group (Figure 1D and Figure S1A–I, Supplementary Table S3). These DVOCs were also divided into 13 categories, among which hydrocarbons, heterocyclic compounds, esters, alcohols, and ketones were the most abundant in the beef cut samples, containing 39, 25, 20, 20, and 14 DVOCs, respectively.

To investigate the flavor characteristics of these beef cuts, we performed a sensory analysis of DVOCs. The results showed that the sensory flavor of DVOCs varied among different groups (Figure 1E,F and Figure S1A–I). For instance, the sweet aroma was highly enriched in the chuck and neck group, followed by the longissimus lumborum and neck group, and the neck and tenderloin group, respectively. The fruity aroma was highly enriched in the longissimus lumborum and rump group. These findings indicate that these flavors may be compounds potentially responsible for the aroma differences among beef cuts.

### 3.3. Comparative Lipidomic Analysis of the Different Beef Cuts

**Figure 2:**
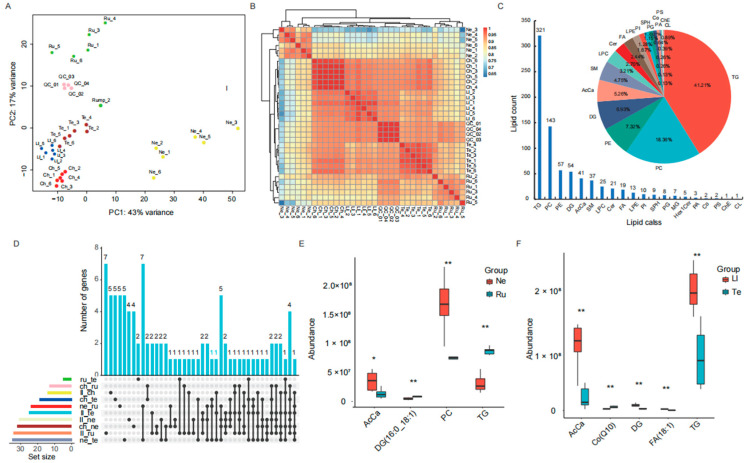
The profile of lipidomic in the five beef cuts. (**A**) PCA of lipid molecules data for quality control samples and all beef cuts. (**B**) Unbiased hierarchical clustering heat map for quality control samples and all beef cuts. (**C**) Percentages of lipid molecules based on chemical taxonomy: subclasses. (**D**) A Venn diagram illustrating the unique and shared DLMs among the ten groups. (**E**) DLMs compare bar charts in the Ne vs. Ch group. (**F**) DLMs compare bar charts in the Ll vs. Te group. * *p* < 0.05, ** *p* < 0.01.

To delineate the dynamic changes in lipidomic profiling, we investigated lipid molecules among various beef cuts. The PCA scores and heatmaps revealed that the quality control (QC) samples were clustered together, indicating the stability of the data and analytical approach applied (Figure 2A,B). Notably, the neck samples were distinctly separated from the other beef cut samples, hinting at unique lipid characteristics among the five different beef cuts (Figure 2B). To further dissect the lipid molecules responsible for the differences in meat quality, 779 lipids were divided into 20 lipid subclasses. The relative abundances of each lipid subclass in the beef cut samples are illustrated in Figure 2C. The triglyceride (TG) class exhibited the highest relative response in beef cut, accounting for 41.21%, followed by phosphatidylcholine (PC, 18.36%) phosphatidylethanolamine (PE, 7.32%), and diacylglycerols (DG, 6.93%).

To explore the potential difference in lipid molecules, we evaluated the lipid alterations among five beef cuts. Using VIP values ≥ 1, *p* values < 0.05, and FC ≥ 2 or FC ≤ 0.5, we detected a total of 95 different lipid molecules (DLMs) among the ten groups. The number of unique DLMs ranged from two in the rump and tenderloin group to seven in the longissimus lumborum and rump group (Figure 2D). Additionally, the number of DLMs varied from five in the rump and tenderloin group to 35 in the neck and tenderloin group (Supplementary Table S4). These DLMs were classified into nine lipid subclasses, including acylcarnitines (AcCas), Cos, DGs, fatty acids (FAs), lysophosphatidylcholines (LPCs), PCs, PEs, sphingomyelins (SMs), and TGs. Notably, TGs accounted for 58.95% of the total DLMs, whereas AcCas and PCs accounted for 13.68% and 13.68%, respectively. We found that the abundance of lipid subclass varied among ten groups (Figure 2E,F and Figure S2A–H). For instance, the abundances of TGs significantly differed among the ten groups, except for the longissimus lumborum and chuck group. AcCas, DG(16:0_18:1), PCs, and TGs also significantly differed between the neck and rump (Figure 2E). AcCas, Co(Q10), DGs, FA(C18:1), and TGs significantly differed between longissimus lumborum and tenderloin (Figure 2F). Furthermore, functional enrichment analysis suggested that the identified DLMs were involved in α-linolenic acid metabolism (bta00592), arachidonic acid metabolism (bta00590), linoleic acid metabolism (bta00591), retrograde endocannabinoid signaling (bta04723), and glycerophospholipid metabolism (bta00564) (Supplementary Table S5).

Furthermore, the ROC curve was conducted for DLMs to identify potential lipid molecular markers among beef cuts. The results revealed that a total of 60 DLMs were candidate lipid markers, which were classified as AcCas, Cos, DGs, FAs, PCs, PEs, and TGs (Supplementary Table S6). Among them, lipid markers in the TG subclass accounted for 73.33% of the total potential markers, while the AcCa and DG subclasses accounted for 8.33% and 8.33%, respectively. We found that the number of lipid markers varied among the ten groups, ranging from one marker in three groups, including the chuck and rump group, the longissimus lumborum and chuck group, and the rump and tenderloin group, to 25 in the neck and tenderloin group (Supplementary Table S6). For instance, FA(18:1) was abundant in longissimus lumborum and emerged as a promising lipid molecular marker within the longissimus lumborum and tenderloin group. Similarly, PC(40:5) emerged as a potential lipid molecular marker for the neck and rump group. Importantly, a range of triglycerides, including TG(18:0_16:1_18:1), TG(18:4_14:1_16:1), TG(19:2_18:1_17:2), TG(19:4_14:1_16:1), TG(20:5_14:1_16:1), and TG(36:0_18:1) were identified as possible molecular markers of lipid variation in the chuck and tenderloin group.

### 3.4. Transcriptomic Analysis Among Five Different Beef Cuts

**Figure 3:**
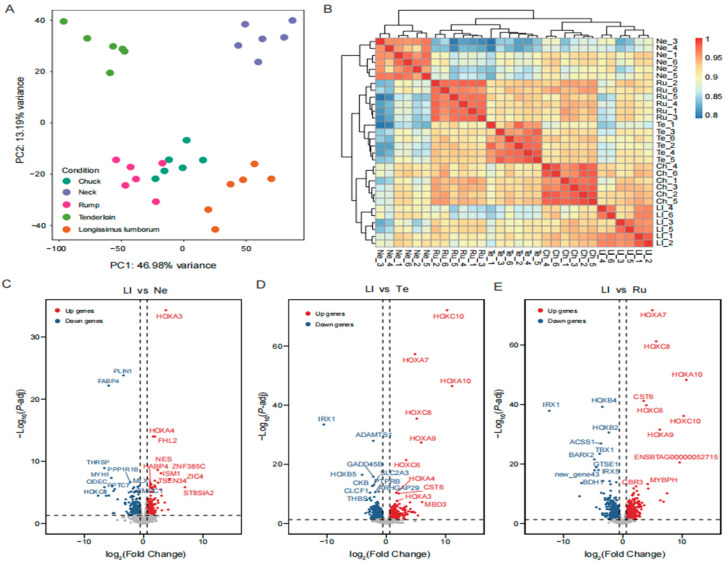
The profile of gene expression in the five beef cuts. (**A**) PCA of gene expression data for all beef cuts. (**B**) Unbiased hierarchical clustering heat map for 9133 genes. (**C**–**E**) The Volcano plot from Ll vs. Ne, Ll vs. Te, and Ll vs. Ru groups, respectively; the other comparisons are shown in the Additional file Figure S3. The *x*-axis represents the log_2_(Fold Change) value, while the *y*-axis depicts the −log_10_(*p*-adj) value. Down-regulated genes are denoted by blue nodes, and up-regulated genes are indicated by red nodes.

To investigate expression profiling of meat quality, we analyzed the transcriptomes of the five different beef cuts. PCA indicated a 60.17% variance, with the first and second components accounting for 46.98% and 13.19%, respectively (Figure 3A). Hierarchical clustering analysis revealed that tenderloin, rump, and chuck were closely related, as these tissues presented similar transcriptome patterns in terms of meat quality (Figure 3B). Intriguingly, the neck cut aggregated into a cluster that was separate from the other cuts. The findings indicated that there were variations in gene expression patterns among the five distinct beef cuts.

To uncover potential differences in gene expression among various beef cuts, we analyzed transcriptome changes and identified DEGs between them. The DEGs containing up-regulated and down-regulated genes were shown in the ten groups, respectively (Figure 3C–E and Figure S3A–G). We identified a total of 4780 DEGs, ranging from 93 in the rump and tenderloin group (47 up-regulated and 46 down-regulated) to 3362 in the neck and rump group (1674 up-regulated and 1688 down-regulated) (Supplementary Table S7). Functional enrichment analysis revealed that the identified DEGs were involved in metabolic pathways (bta01100), thermogenesis (bta04714), fatty acid metabolism (bta01212), oxidative phosphorylation (bta00190), and regulation of actin cytoskeleton (bta04810).

### 3.5. Integrated Analysis of Differential Metabolism and Gene Expression

**Figure 4:**
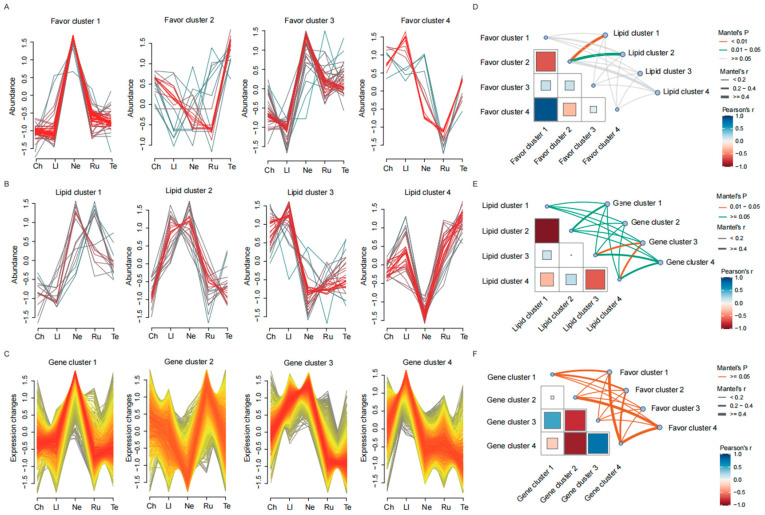
The k-means cluster analysis of metabolites and gene expression. (**A**–**C**) The k-means cluster analysis of DVOCs (**A**), DLMs (**B**), and DEGs (**C**). The *x*-axis depicts samples from five representative beef cuts, and the *y*-axis depicts the abundance per DVOC and DLM, as well as expression change for DEG. (**D**–**F**) The correlation analysis of DVOCs, DLMs, and DEGs among clusters. The correlation analysis between the flavor compound cluster and lipid molecule cluster (**D**). The correlation analysis between lipid molecule cluster and gene expression cluster (**E**). The correlation analysis between the gene expression cluster and flavor compound cluster (**F**).

To gain further insight into metabolites and gene expression pattern changes among different beef cuts, each one of the 139 DVOCs, 95 DLMs, and 4780 DEGs previously identified was divided into four clusters based on the accumulation patterns using the k-means clustering algorithm (Figure 4A–C). We observed that the flavor compounds in cluster 1 and cluster 3 were predominantly enriched in the neck cut, while those in cluster 4 were accumulated in the longissimus lumborum cut. The lipid molecules in cluster 2 were enriched in the longissimus lumborum and neck cut, whereas the lipid molecules in cluster 4 were less accumulated in the neck cut. Meanwhile, gene cluster 2 was enriched in the longissimus lumborum and rump cut, but less abundant in the neck cut. To better understand the relationship between metabolite accumulation and gene expression patterns, we then performed correlation analysis among the clusters with the Mantel test. Our results revealed that flavor cluster 2 was significantly correlated with lipid cluster 1 (r = 0.79, *p* = 0.008) and lipid cluster 2 (r = 0.60, *p* = 0.02) (Figure 4D). Additionally, gene cluster 3 was significantly associated with lipid cluster 3 (r = 0.55, *p* = 0.04) and lipid cluster 4 (r = 0.76, *p* = 0.02) (Figure 4E).

**Figure 5:**
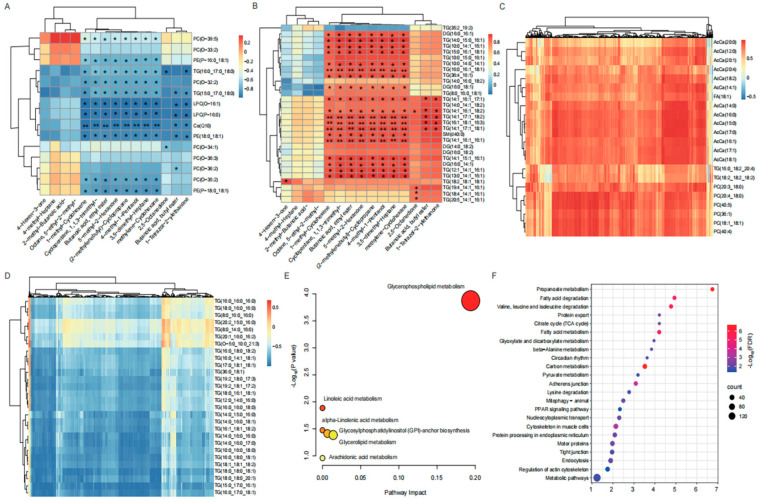
Correlation and functional analysis of differential compounds and genes associated with meat quality trait. (**A**) Correlation analysis of DVOCs in flavor cluster 2 and DLMs in lipid cluster 1. (**B**) Correlation analysis of DVOCs in flavor cluster 2 and DLMs in lipid cluster 2. (**C**) Correlation analysis of DEGs in gene cluster 3 and DLMs in lipid cluster 3. (**D**) Correlation analysis of DEGs in gene cluster 3 and DLMs in lipid cluster 4. (**E**) Pathway enrichment analysis for DLMs in lipid cluster 1 and lipid cluster 2. (**F**) Pathway enrichment analysis for DEGs in gene cluster 3. The abscissa is the log_2_(FDR) value, and the ordinate is the pathway term. * *p* < 0.05, ** *p* < 0.01.

To further explore the relationship between DVOCs, DLMs, and DEGs within the candidate clusters, we performed a Pearson correlation analysis. The results revealed that eight DVOCs in flavor cluster 2 (2-methylenebutyl-Cyclopropane, 5-methyl-2-Hexanone, 4-methyl-1-Pentanol, Butanoic acid-ethyl ester, 3,5-dimethyl-Heptane, methylene-Cyclohexane, Cyclopentane, 1,1,3-trimethyl-, and 1-methyl-Cyclohexene) were negatively correlated with 11 DLMs in lipid cluster 1 (Co(Q10), LPC(O-16:1), LPC(P-16:0), PC(O-39:5), PC(O-32:2), PC(O-35:2), PE(18:0_18:1), PE(P-16:0_18:1), PE(P-18:0_18:1), TG(15:0_17:0_18:0), and TG(16:0_17:0_18:0)) (Figure 5A). Butanoic acid, ethyl ester, and 1-tetrazol-2-ylethanone were also negatively correlated with five DLMs (Co(Q10), TG(15:0_17:0_18:0), LPC(O-16:1), LPC(P-16:0), and PE(18:0_18:1)). Interestingly, these same eight DVOCs were positively correlated with 19 DLMs in lipid cluster 2 (Figure 5B). The butanoic acid, ethyl ester, and 1-tetrazol-2-ylethanone were also positively correlated with five DLMs (TG(14:1_16:1_17:1), TG(14:1_16:1_18:2), TG(14:1_16:1_18:2), TG(16:1_18:1_16:3), and TG(14:1_17:1_18:1)). The DEGs in gene cluster 3 were positively correlated with the DLMs in lipid cluster 3 but negatively correlated with the DLMs in lipid cluster 4 (Figure 5C,D). Functional enrichment analysis of lipid clusters 1 and 2 suggested that the identified DLMs were involved in glycerophospholipid metabolism (bta00564) and glycerolipid metabolism (bta00561), respectively (Figure 5E, Supplementary Table S7). For gene cluster 3, the DEGs were involved mainly in propanoate metabolism (bta00640), valine, leucine, and isoleucine degradation (bta00280), fatty acid degradation (bta00071), and fatty acid metabolism (bta01212), among others (Figure 5F). These findings indicate that the gene expression profiles are associated with the metabolic pathways in various beef cuts.

### 3.6. Identification of Potential Genes Regulating the Metabolic Network for Meat Quality

**Figure 6:**
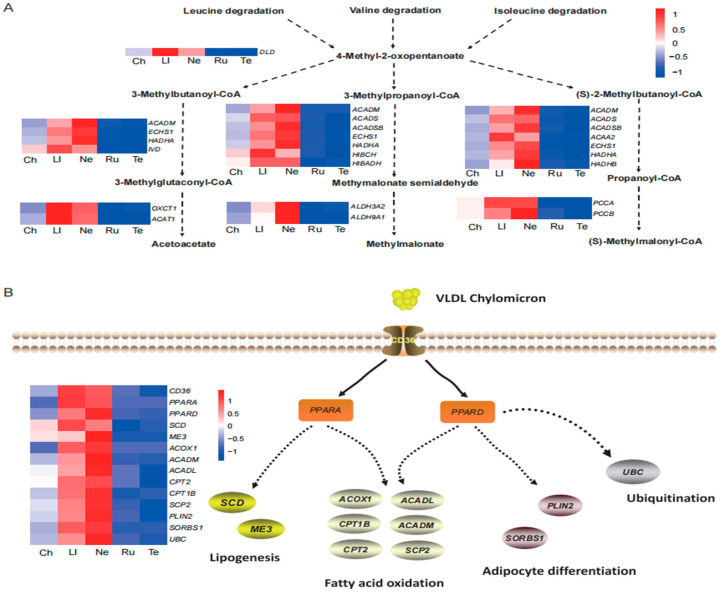
The important pathways associated with five beef cuts in cattle. (**A**) The valine, leucine, and isoleucine degradation pathways contribute to the meat quality among beef cuts. (**B**) The lipid metabolism pathways contribute to the meat quality among beef cuts.

Amino acids and lipids, as crucial components of meat flavor precursors, significantly impact the formation of meat quality traits. Utilizing the clustering data, we detected 19 DEGs involved in the valine, leucine, and isoleucine degradation pathways (Figure 6A). Specifically, the DLD gene was involved in 4-Methyl-2-oxopentanoate degradation. Furthermore, *ACADM*, *ECHS1*, *HADHA*, *IVD*, *OXCT1*, and *ACAT1* play essential roles in the formation of acetoacetate. Additionally, *ACADM*, *ACADS*, *ACADSB*, *ACAA2*, *ECHS1*, *HADHA/B*, HIBCH, and HIBADH affect the formation of methymalonate semialdehyde and propanoyl-CoA. *ALDH3A2* and *ALDH9A1* regulate methylmalonate, whereas *PCCA* and *PCCB* may be indispensable for (S)-methylmalonyl-CoA (Figure 6A). To further explore the potential key genes involved in lipid metabolism pathways, we obtained 14 critical DEGs from gene cluster 3. Among them, *SCD* and *ME3* were involved in lipogenesis. *ACOX1*, *ACADL*, *CPT1B*, *ACADM*, *CPT2*, and *SCP2* play pivotal roles in fatty acid oxidation. *PLIN2* and *SORBS1* regulate adipocyte differentiation, while *CD36*, *PPARA*, and *PPARD* may be crucial for fatty acid transport in beef cattle (Figure 6B). Notably, all these 33 genes were specifically highly expressed in the longissimus lumborum and neck cuts and lowly expressed in the rump and tenderloin cuts (Figure 6A,B). The varying expression patterns of these genes could potentially influence the development of meat quality across different beef cuts.

## 4. Discussion

The growing consumer demand for higher quality, healthier meat products, coupled with the perception that beef is one of the most nutritious meat sources, has led to the growing popularity of beef [26]. However, with the improvement in living standards, consumers’ expectations of beef are no longer limited to the nutritional level, and they are also increasingly focused on the sensory quality of beef, including taste, flavor, tenderness, and juiciness. This trend requires the industry to ensure that beef products meet consumer expectations regarding quality and health benefits through improved production practices. Therefore, scientific research exploring effective ways to improve the quality and nutritional value of beef is urgently needed. To meet this demand, we performed a comparative study based on integrated flavoromic, lipidomic, and transcriptomic analyses on different beef cuts from Chinese Wagyu cattle.

A total of 240 VOCs were identified among beef cut samples, where hydrocarbons, heterocyclic compounds, and esters were more dominant. This finding was consistent with previous studies carried out on raw beef [27]. We also identified 139 DVOCs using strict VIP value, *p* value, and FC criteria. Among the 25 heterocyclic DVOCs, six had odor characteristics. For example, tetrahydro-2,2-dimethyl-5-(1-methyl-1-propenyl)-furan is associated with a sweet and minty aroma. As important volatile substances, a total of 20 ester compounds were identified, half of which had multiple odor characteristics. For instance, 2-butoxyethyl acetate, 2(5H)-furanone, 3-hydroxy-4,5-dimethyl butanoic acid, and 3-methylbutyl ester have a sweet aroma [28,29], while butanoic acid, butyl ester, butanoic acid ethyl ester, propanoic acid hexyl ester, and acetic acid pentyl ester are associated with a fruity aroma [30,31]. However, since the GC approach was limited primarily to volatile and semi-volatile compound detection, further metabolomic analyses could provide novel insight into beef compounds not analyzed in the present study.

As important metabolites, lipids are the main flavor precursors in meat and have a direct effect on beef flavor [32]. Based on the untargeted lipidomic analysis, we identified 779 lipid molecules among the beef samples, where TGs, PCs, PEs, and DGs accounted for 73.82% of lipids in the beef samples, which was similar to previous reports on beef [33]. TGs and DGs, as major lipid species, are primarily responsible for energy storage, and their decomposition products include a variety of polyunsaturated fatty acids that are important for human health [34]. PCs and PEs are phospholipids, and their metabolites can participate in cell signal transmission as secondary messengers and play important roles in life activities [35]. We also identified 95 DLMs among the ten groups, 60 of which are candidate lipid markers. For example, FA(18:1), also known as oleic acid, is a monounsaturated fatty acid (MUFA) that affects human health by improving the immune system and atherosclerosis, decreasing the risk of atherosclerosis, and preventing cardiovascular disease [36]. PC(40:5) is a key component of the cellular lipid bilayer and is involved in metabolism and signaling [37]. These lipid biomarkers have value in identifying the source of beef cuts.

Based on the analysis of the metabolic patterns across various beef cuts, we determined that the 139 DVOCs, 95 DLMs, and 4780 DEGs could each be categorized into four distinct clusters. Leveraging this clustered dataset, we were able to delve into the metabolic regulatory networks and identify the associated candidate markers and genes. The flavor compounds from clusters 1 and 3 accumulated in the neck muscle, in which most of the beef-cut aromatics (octahydro-1H-indene) and hydrocarbons (1-methoxyhexane) were included. Basically, 1-methoxy-hexane is a derivative of hydrocarbon widely existing in the eukaryotic cell. Research has revealed that the accumulation of reactive oxygen species can lead to the production of hydrocarbons, and the peroxidation of polyunsaturated fatty acids in meat can lead to the production of straight-chain alkanes [38]. The lipid molecules in cluster 2 were abundant in both the longissimus lumborum and neck muscles, with significant fractions being composed of TGs. Moreover, correlation analysis revealed that 15 DLMs in the TG subclass were positively correlated with eight DVOCs (Figure 5B). For example, TG(14:1_16:1_16:1), a dipalmitoleic acid triglyceride, plays an important role in metabolism as an energy source and transporter of dietary fat [39]. Therefore, the volatile and lipid profiles of the five different beef cuts were significantly different. These disparities may contribute to the specific flavors of each cut and allow them to be effectively distinguished.

The process of meat quality formation involves the biosynthesis of characteristic metabolites. Lipids and amino acids are indispensable components as key indicators of meat quality [33]. In gene cluster 3, differences in the expression levels of 19 genes related to leucine and isoleucine degradation were detected across different beef cuts (Figure 6A). For example, the *DLD* gene was highly expressed in the longissimus lumborum muscle, and involved in regulating the degradation of valine, leucine, and isoleucine, respectively [40]. The *IVD* gene was strongly correlated with amino acids and intramuscular fat [14], and is highly expressed in the longissimus lumborum and neck muscles. Additionally, *PCCA* and *PCCB* regulate the catabolism of propionyl-CoA to produce (S)-methylmalonyl-CoA [41]. Furthermore, 14 DEGs associated with lipid metabolic pathways exhibited variations across the different beef cuts. For instance, *CD36* encodes a scavenger receptor that plays a key role in fatty acid transport and is involved in lipid accumulation and metabolic functions [42]. The *SCD* serves as a pivotal enzyme in the lipogenesis process, and is responsible for regulating and catalyzing the conversion of saturated fatty acids (SFAs) to MUFAs [43]. Fatty acid β-oxidation is an important reaction in the lipid metabolism pathway. Six candidate genes (*ACOX1*, *ACADL*, *CPT2*, *CPT1B*, *SCP2*, and *ACADM*) were found to be involved in regulating fatty acid β-oxidation in the present study (Figure 6B). *ACOX1* was highly expressed in the longissimus lumborum and neck tissues, with log_2_(FC) values of 2.2 and 2.4, respectively. *ACOX1* is involved in the desaturation of acyl-CoAs to 2-trans-enoyl-CoAs and catabolizes very long-chain fatty acids (VLCFAs) [44]. As a crucial component in mitochondrial long-chain fatty acid oxidation, *CPT2* governs the entry of long-chain fatty acids into the mitochondria and their subsequent conjugation with CoA for metabolism, ultimately yielding energy [45]. Moreover, *CPT1B* plays a regulatory role in fatty acid transport [46]. *ACADM* was highly expressed in the neck muscle with a log_2_(FC) value of 7.16. This gene encodes medium-chain acyl-CoA dehydrogenase, which catalyzes the initial step in the mitochondrial β-oxidation of medium-chain acyl-CoA metabolism [47]. Taken together, the differences in meat quality among the beef cuts were regulated by a complex network. Despite the relatively small dataset, promising results were obtained that can serve as an initial step in implementing sensitive tools like flavoromic, lipidomic, and transcriptomic profiles in the evaluation of beef quality in Chinese Wagyu cattle as well as in other cattle breeds.

## 5. Conclusions

This study used GC-MS, LC-HRMS, and RNA-seq to analyze and identify the chuck, neck, rump, tenderloin, and longissimus lumborum cuts of Chinese Wagyu cattle. Across these various beef cuts, a total of 240 VOCs and 779 LMs were identified, with hydrocarbons and triglycerides emerging as the most prominent compounds, respectively. The sweet and fruity aroma compounds played pivotal roles in shaping the distinct aroma profiles among the different beef cuts. Furthermore, a total of 60 key lipid molecular markers were pinpointed as playing crucial roles in the generation of essential lipid compounds across five different beef cuts. Integration analysis showed that the valine, leucine, and isoleucine degradation pathways and lipid metabolism were the key pathways among beef cuts. This study has identified key metabolites and candidate genes that influence meat quality across different beef cuts, providing a theoretical basis for further research endeavors aimed at enhancing meat quality traits in beef cattle breeding.

## Data Availability

The data presented in this study are available in the article and Supplementary Materials.

## Supplementary Materials

The following supporting information can be downloaded at: https://www.mdpi.com/article/10.3390/foods14050716/s1, Figure S1: The identification of differential volatile organic compounds (DVOCs) among the five beef cuts; Figure S2: Comparative analysis of DLMs between groups; Figure S3: Comparative analysis of DEGs between groups. Supplementary Dataset S1: The statistical flavor compounds in chuck, neck, rump tenderloin, and longissimus lumborum cuts; Supplementary Dataset S2: The statistical lipid molecules in chuck, neck, rump tenderloin, and longissimus lumborum cuts; Supplementary Dataset S3: The statistical gene expression values (FPKM) of chuck, neck, rump tenderloin, and longissimus lumborum cuts; Supplementary Table S1: Reads mapping summary of chuck, neck, rump tenderloin, and longissimus lumborum cuts; Supplementary Table S2. Orthogonal partial least squares discriminant analysis (OPLS-DA) model validation; Supplementary Table S3. Analysis of differential volatile organic compounds (DVOCs) between chuck, neck, rump tenderloin, and longissimus lumborum cuts; Supplementary Table S4. Analysis of different lipid molecules (DLMs) between chuck, neck, rump tenderloin, and longissimus lumborum cuts; Supplementary Table S5. Function enrichment Analysis of DLMs between chuck, neck, rump tenderloin, and longissimus lumborum cuts; Supplementary Table S6. Candidate lipid molecular markers among ten groups; Supplementary Table S7. Analysis of differentially expressed genes (DEGs) between chuck, neck, rump tenderloin, and longissimus lumborum cuts.

## Abbreviations

The following abbreviations are used in this manuscript:

IMF	Intramuscular fat content
VOCs	Volatile organic compounds
DVOCs	Differential volatile organic compounds
DLMs	Differential lipid molecules
DEGs	Differentially expressed genes

## References

[1] Huang Y., Cao D., Chen Z., Chen B., Li J., Guo J., Dong Q., Liu L., Wei Q. (2021). Red and processed meat consumption and cancer outcomes: Umbrella review. Food Chem..

[2] Geletu U.S., Usmael M.A., Mummed Y.Y., Ibrahim A.M. (2021). Quality of Cattle Meat and Its Compositional Constituents. Vet. Med. Int..

[3] Hou X., Zhang R., Yang M., Niu N., Wu J., Shu Z., Zhang P., Shi L., Zhao F., Wang L. (2023). Metabolomics and lipidomics profiles related to intramuscular fat content and flavor precursors between Laiwu and Yorkshire pigs. Food Chem..

[4] Lebedová N., Bureš D., Needham T., Fořtová J., Řehák D., Bartoň L. (2022). Histological composition, physiochemical parameters, and organoleptic properties of three muscles from Fleckvieh bulls and heifers. Meat Sci..

[5] Ma Q., Kou X., Yang Y., Yue Y., Xing W., Feng X., Liu G., Wang C., Li Y. (2023). Comparison of Lipids and Volatile Compounds in Dezhou Donkey Meat with High and Low Intramuscular Fat Content. Foods.

[6] Khan M.I., Jo C., Tariq M.R. (2015). Meat flavor precursors and factors influencing flavor precursors—A systematic review. Meat Sci..

[7] Shakoor A., Zhang C., Xie J., Yang X. (2022). Maillard reaction chemistry in formation of critical intermediates and flavour compounds and their antioxidant properties. Food Chem..

[8] Jung E.Y., Hwang Y.H., Joo S.T. (2016). The Relationship between Chemical Compositions, Meat Quality, and Palatability of the 10 Primal Cuts from Hanwoo Steer. Korean J. Food Sci. Anim. Resour..

[9] Picard B., Gagaoua M. (2020). Muscle Fiber Properties in Cattle and Their Relationships with Meat Qualities: An Overview. J. Agric. Food Chem..

[10] Ke Y., Mitacek R.M., Abraham A., Mafi G.G., VanOverbeke D.L., DeSilva U., Ramanathan R. (2017). Effects of Muscle-Specific Oxidative Stress on Cytochrome c Release and Oxidation-Reduction Potential Properties. J. Agric. Food Chem..

[11] Yu Q., Tian X., Sun C., Shao L., Li X., Dai R. (2019). Comparative transcriptomics to reveal muscle-specific molecular differences in the early postmortem of Chinese Jinjiang yellow cattle. Food Chem..

[12] Giovanini de Oliveira Sartori A., Silva Antonelo D., Ribeiro G.H., Colnago L.A., de Carvalho Balieiro J.C., Francisquine Delgado E., Contreras Castillo C.J. (2024). Lipidome and metabolome profiling of longissimus lumborum beef with different ultimate pH and postmortem aging. Meat Sci..

[13] Zhang T., Niu Q., Wang T., Zheng X., Li H., Gao X., Chen Y., Gao H., Zhang L., Liu G.E. (2022). Comparative Transcriptomic Analysis Reveals Diverse Expression Pattern Underlying Fatty Acid Composition among Different Beef Cuts. Foods.

[14] Zhang T., Wang T., Niu Q., Zheng X., Li H., Gao X., Chen Y., Gao H., Zhang L., Liu G.E. (2022). Comparative transcriptomic analysis reveals region-specific expression patterns in different beef cuts. BMC Genom..

[15] Pang Z., Xu L., Viau C., Lu Y., Salavati R., Basu N., Xia J. (2024). MetaboAnalystR 4.0: A unified LC-MS workflow for global metabolomics. Nat. Commun..

[16] Zhou Z., Shen X., Chen X., Tu J., Xiong X., Zhu Z.J. (2019). LipidIMMS Analyzer: Integrating multi-dimensional information to support lipid identification in ion mobility-mass spectrometry based lipidomics. Bioinformatics.

[17] Thévenot E.A., Roux A., Xu Y., Ezan E., Junot C. (2015). Analysis of the Human Adult Urinary Metabolome Variations with Age, Body Mass Index, and Gender by Implementing a Comprehensive Workflow for Univariate and OPLS Statistical Analyses. J. Proteome Res..

[18] Zhang T., Wang T., Niu Q., Xu L., Chen Y., Gao X., Gao H., Zhang L., Liu G.E., Li J. (2022). Transcriptional atlas analysis from multiple tissues reveals the expression specificity patterns in beef cattle. BMC Biol..

[19] Chen S., Zhou Y., Chen Y., Gu J. (2018). Fastp: An ultra-fast all-in-one FASTQ preprocessor. Bioinformatics.

[20] Kim D., Langmead B., Salzberg S.L. (2015). HISAT: A fast spliced aligner with low memory requirements. Nat. Methods.

[21] Li H., Handsaker B., Wysoker A., Fennell T., Ruan J., Homer N., Marth G., Abecasis G., Durbin R. (2009). The Sequence Alignment/Map format and SAMtools. Bioinformatics.

[22] Pertea M., Kim D., Pertea G.M., Leek J.T., Salzberg S.L. (2016). Transcript-level expression analysis of RNA-seq experiments with HISAT, StringTie and Ballgown. Nat. Protoc..

[23] Love M.I., Huber W., Anders S. (2014). Moderated estimation of fold change and dispersion for RNA-seq data with DESeq2. Genome Biol..

[24] Conway J.R., Lex A., Gehlenborg N. (2017). UpSetR: An R package for the visualization of intersecting sets and their properties. Bioinformatics.

[25] Sherman B.T., Hao M., Qiu J., Jiao X., Baseler M.W., Lane H.C., Imamichi T., Chang W. (2022). DAVID: A web server for functional enrichment analysis and functional annotation of gene lists (2021 update). Nucleic Acids Res..

[26] Caputo V., Sun J., Staples A.J., Taylor H. (2024). Market outlook for meat alternatives: Challenges, opportunities, and new developments. Trends Food Sci. Technol..

[27] Bhadury D., Nolvachai Y., Marriott P.J., Tanner J., Tuck K.L. (2021). Detection of Volatiles from Raw Beef Meat from Different Packaging Systems Using Solid-Phase Microextraction GC-Accurate Mass Spectrometry. Foods.

[28] Zhang Z., Blank I., Wang B., Cao Y. (2022). Changes in odorants and flavor profile of heat-processed beef flavor during storage. J. Food Sci..

[29] Wang H., Yang P., Liu C., Song H., Pan W., Gong L. (2022). Characterization of key odor-active compounds in thermal reaction beef flavoring by SGC×GC-O-MS, AEDA, DHDA, OAV and quantitative measurements. J. Food Compos. Anal..

[30] Liu Q., Gu X., Wen R., Sun C., Yu Q. (2024). Changes in meat quality and volatile flavor compounds profile in beef loin during dry-aging. LWT.

[31] SÁ A.G.A., Meneses A.C.d., Araújo P.H.H.d., Oliveira D.d. (2017). A review on enzymatic synthesis of aromatic esters used as flavor ingredients for food, cosmetics and pharmaceuticals industries. Trends Food Sci. Technol..

[32] Fu Y., Cao S., Yang L., Li Z. (2022). Flavor formation based on lipid in meat and meat products: A review. J. Food Biochem..

[33] Zhou L., Ren Y., Shi Y., Fan S., Zhao L., Dong M., Li J., Yang Y., Yu Y., Zhao Q. (2024). Comprehensive foodomics analysis reveals key lipids affect aroma generation in beef. Food Chem..

[34] Han X., Ye H. (2021). Overview of Lipidomic Analysis of Triglyceride Molecular Species in Biological Lipid Extracts. J. Agric. Food Chem..

[35] Momchilova A., Markovska T. (1999). Phosphatidylethanolamine and phosphatidylcholine are sources of diacylglycerol in ras-transformed NIH 3T3 fibroblasts. Int. J. Biochem. Cell Biol..

[36] Poleti M.D., Regitano L.C., Souza G.H., Cesar A.S., Simas R.C., Silva-Vignato B., Montenegro H., Pértille F., Balieiro J.C., Cameron L.C. (2020). Proteome alterations associated with the oleic acid and cis-9, trans-11 conjugated linoleic acid content in bovine skeletal muscle. J. Proteomics..

[37] Li X., Nakayama K., Goto T., Kimura H., Akamatsu S., Hayashi Y., Fujita K., Kobayashi T., Shimizu K., Nonomura N. (2021). High level of phosphatidylcholines/lysophosphatidylcholine ratio in urine is associated with prostate cancer. Cancer Sci..

[38] Liu M., Li Y., Wang G., Guo N., Liu D., Li D., Guo L., Zheng X., Yu K., Yu K. (2019). Release of volatile organic compounds (VOCs) from colorectal cancer cell line LS174T. Anal Biochem..

[39] Guan M., Dai D., Li L., Wei J., Yang H., Li S., Zhang Y., Lin Y., Xiong S., Zhao Z. (2017). Comprehensive qualification and quantification of triacylglycerols with specific fatty acid chain composition in horse adipose tissue, human plasma and liver tissue. Talanta.

[40] Lavorato M., DIadarola D., Remes C., Kaur P., Broxton C., Mathew N.D., Xiao R., Seiler C., Nakamaru-Ogiso E., Anderson V.E. (2024). dldhcri3 zebrafish exhibit altered mitochondrial ultrastructure, morphology, and dysfunction partially rescued by probucol or thiamine. JCI Insight.

[41] He W., Marchuk H., Koeberl D., Kasumov T., Chen X., Zhang G.F. (2024). Fasting alleviates metabolic alterations in mice with propionyl-CoA carboxylase deficiency due to Pcca mutation. Commun. Biol..

[42] Hao J.W., Wang J., Guo H., Zhao Y.Y., Sun H.H., Li Y.F., Lai X.Y., Zhao N., Wang X., Xie C. (2020). CD36 facilitates fatty acid uptake by dynamic palmitoylation-regulated endocytosis. Nat. Commun..

[43] Liu L., Wang Y., Liang X., Wu X., Liu J., Yang S., Tao C., Zhang J., Tian J., Zhao J. (2020). Stearoyl-CoA Desaturase is Essential for Porcine Adipocyte Differentiation. Int. J. Mol. Sci..

[44] Lu D., He A., Tan M., Mrad M., El Daibani A., Hu D., Liu X., Kleiboeker B., Che T., Hsu F.F. (2024). Liver ACOX1 regulates levels of circulating lipids that promote metabolic health through adipose remodeling. Nat. Commun..

[45] Gonzalez-Hurtado E., Lee J., Choi J., Wolfgang M.J. (2018). Fatty acid oxidation is required for active and quiescent brown adipose tissue maintenance and thermogenic programing. Mol. Metab..

[46] Xiong J. (2018). Fatty Acid Oxidation in Cell Fate Determination. Trends Biochem. Sci..

[47] Ma A.P.Y., Yeung C.L.S., Tey S.K., Mao X., Wong S.W.K., Ng T.H., Ko F.C.F., Kwong E.M.L., Tang A.H.N., Ng I.O. (2021). Suppression of ACADM-Mediated Fatty Acid Oxidation Promotes Hepatocellular Carcinoma via Aberrant CAV1/SREBP1 Signaling. Cancer Res..

