# Supplementary material for: Flavor, Lipid, and Transcriptomic Profiles of Chinese Wagyu Beef Cuts: Insights into Meat Quality Differences
Source: Foods. 2025 Feb 20;14(5):716. doi: 10.3390/foods14050716 (PMC11899191; doi:10.3390/foods14050716)
Supplement: Supplementary file 1 [file foods-14-00716-s001.zip › Additional file Figure Legend----20250217.pdf]

Additional file Figure Legends

Figure S1. The identification of differential volatile organic compounds (DVOCs) among the five beef cuts. DVOCs were detected by Venn diagram based on VIP value, *p*-value, and fold change. Radar map of DVOCs sensory flavor characteristics. The outermost name represents the sensory flavor feature, and the number corresponding to the green dot represents the number of the corresponding sensory flavor feature. Network diagram of sensory flavor characteristics and DVOCs between chuck and neck. Orange circles represent sensory flavor characteristics and turquoise circles represent differential metabolites. A-I represent Ch vs. Ru group, Ch vs. Te group, Ll vs. Ch group, Ll vs. Ne group, Ll vs. Ru group, Ll vs. Te group, Ne vs. Ru group, Ne vs. Te group, and Ru vs. Te group, respectively.

Figure S2. Comparative analysis of DLMs between groups. A-H represent Ch vs. Ne group, Ll vs. Ch group, Ru vs. Te group, Ch vs. Ru group, Ch vs. Te group, Ll vs. Ne group, Ch vs. Ne group, and Ne vs. Te group, respectively. $*p < 0.05$,$**p < 0.01$.

Figure S3. Comparative analysis of DEGs between groups. A-G represent Ll vs. Ch group, Ch vs. Ne group, Ch vs. Ru group, Ch vs. Te group, Ne vs. Ru group, Ne vs. Te group, and Ru vs. Te group, respectively. The abscissa is $\log_2$(Fold Change) value, and the ordinate is $-\log_{10}$(*p*-adj). Blue nodes represent down-regulated genes, red nodes represent up-regulated genes.
