# Supplementary figures and images for: Flavor, Lipid, and Transcriptomic Profiles of Chinese Wagyu Beef Cuts: Insights into Meat Quality Differences
Source: Foods. 2025 Feb 20;14(5):716. doi: 10.3390/foods14050716 (PMC11899191; doi:10.3390/foods14050716)

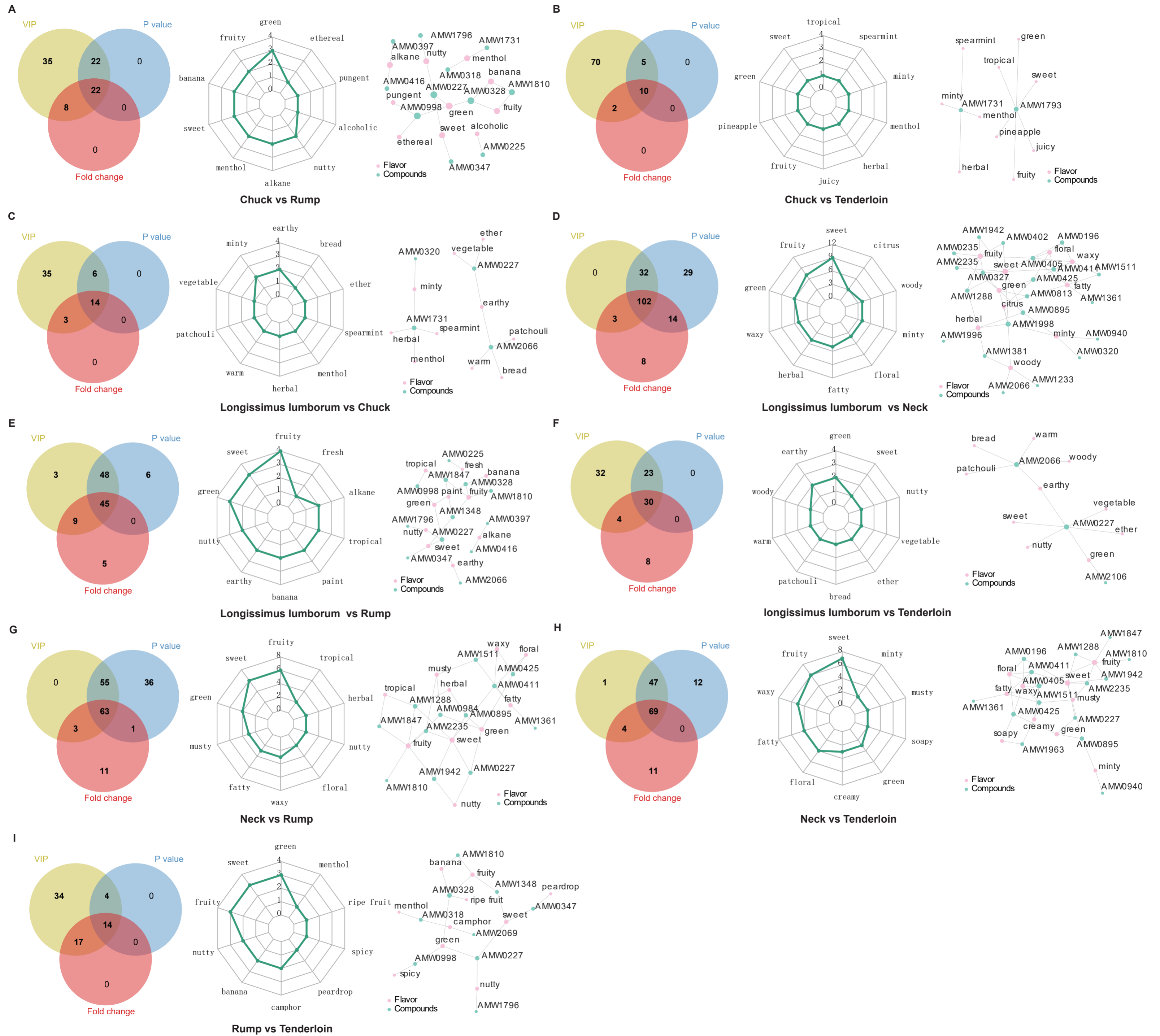

Supplement: Supplementary file 1 [file foods-14-00716-s001.zip › Additional file FigureS1.pdf]

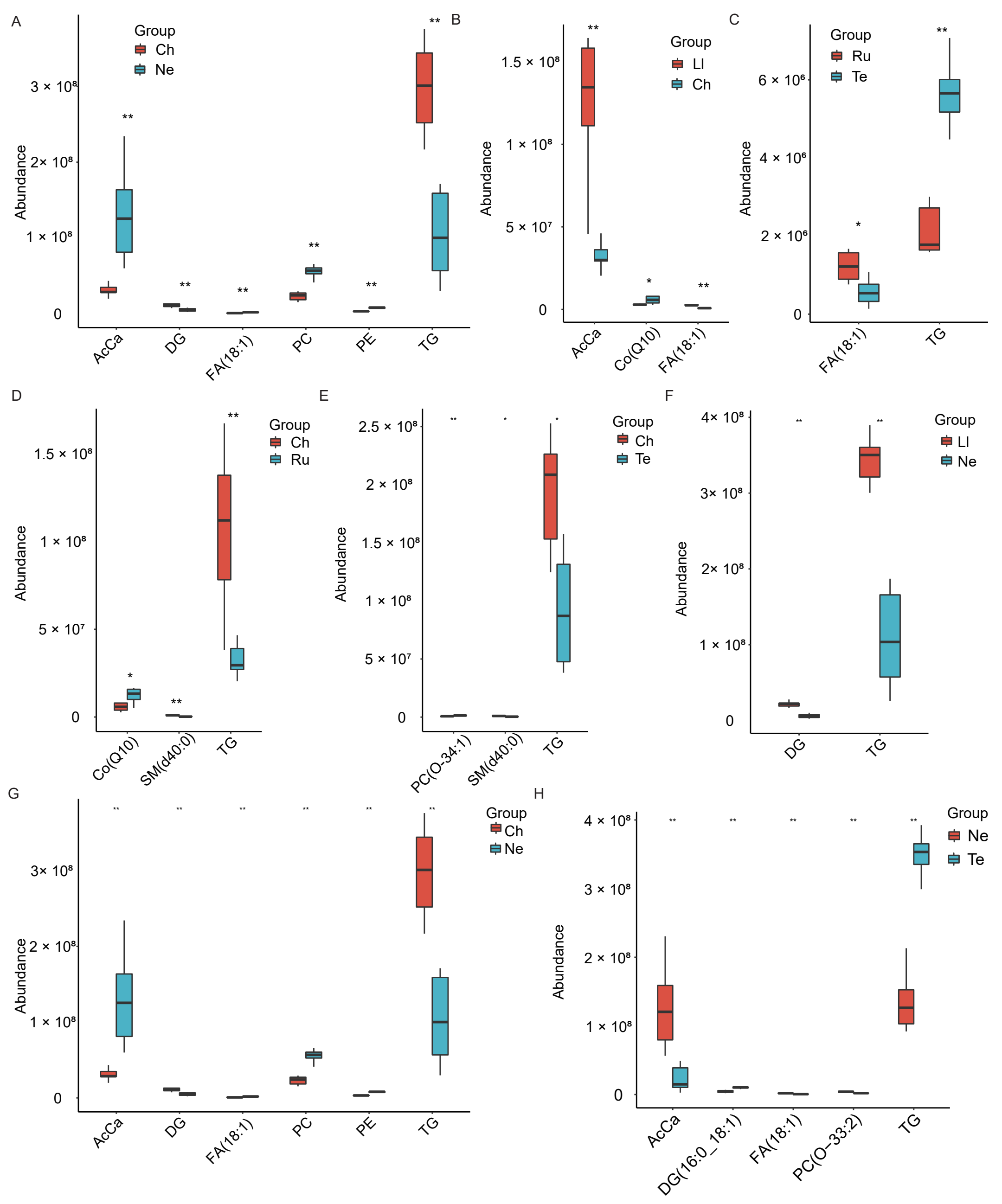

Supplement: Supplementary file 1 [file foods-14-00716-s001.zip › Additional file FigureS2.pdf]
