# Supplementary material for: Flavor, Lipid, and Transcriptomic Profiles of Chinese Wagyu Beef Cuts: Insights into Meat Quality Differences
Source: Foods. 2025 Feb 20;14(5):716. doi: 10.3390/foods14050716 (PMC11899191; doi:10.3390/foods14050716)
Supplement: Supplementary file 1 [file Additional file FigureS3.pdf › Additional file FigureS3.pdf]

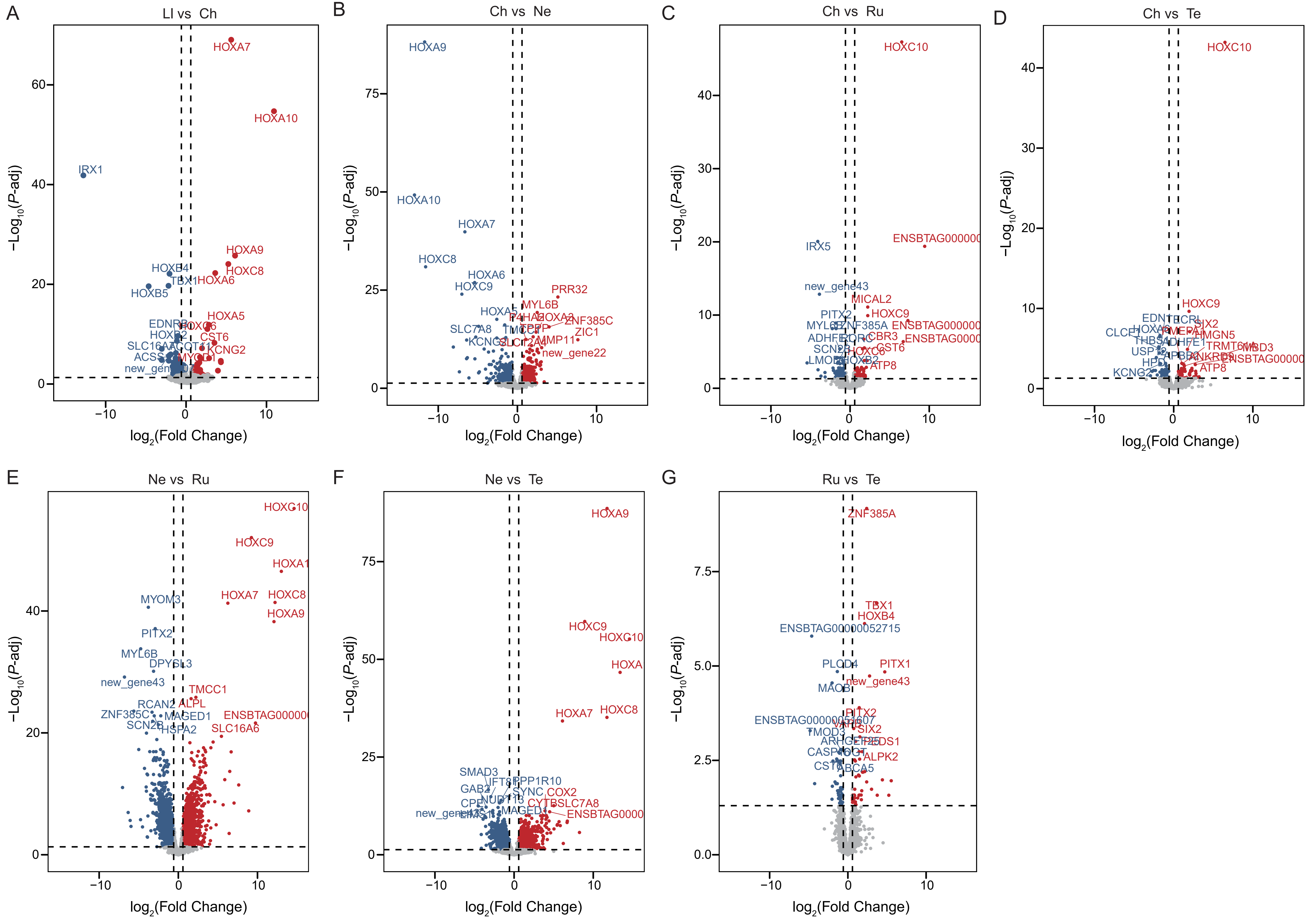
A
Ll vs Ch
HOXA7
HOXA10
IRX1
HOXA9
HOXB4
HOXC8
TBX1
HOXA6
HOXB5
HOXA5
EDNRB
HOXB2
CST6
SLC16A6
KCNG2
ACSS
MYOD1
−Log10(P-adj)
log2(Fold Change)
B
Ch vs Ne
HOXA9
HOXA10
HOXA7
HOXC8
HOXA6
HOXC9
PRR32
MYL6B
HOXA5
ZNF385C
SLC7A8
ZIC1
MMP11
new_gene22
C
Ch vs Ru
HOXC10
ENSBTAG00000
IRX5
new_gene43
MICAL2
PITX2
HOXC9
ZNF385A
CBR3
CST6
ATP8
D
Ch vs Te
HOXC10
HOXC9
SIX2
EDN1
CLCF1
THBS4
AMGN5
USP12
TRMT61B
MBD3
ANKRD9
ENSBTAG00000
ATP8
KCNG2
E
Ne vs Ru
HOXC10
HOXC9
MYOM3
HOXA7
HOXC8
HOXA9
PITX2
MYL6B
DPYSL3
new_gene43
TMCC1
RCAN2
ALPL
ZNF385C
MAGED1
ENSBTAG00000
SCN2B
HSPA2
SLC16A6
F
Ne vs Te
HOXA9
HOXC9
HOXC10
HOXA7
HOXC8
SMAD3
PPP1R10
GAB2
SYNC
COX2
SLC7A8
MAGED1
ENSBTAG0000
G
Ru vs Te
ZNF385A
TBX1
HOXB4
ENSBTAG00000052715
PLOD4
PITX1
new_gene43
MAOB
PITX2
SIX2
TMOD3
ALPK2
CST6
ABCA5
